# Engineered IL-21 Cytokine Muteins Fused to Anti-PD-1 Antibodies Can Improve CD8+ T Cell Function and Anti-tumor Immunity

**DOI:** 10.3389/fimmu.2020.00832

**Published:** 2020-05-08

**Authors:** Shanling Shen, Gail Sckisel, Anupama Sahoo, Almin Lalani, Doug Den Otter, Josh Pearson, Jason DeVoss, Jay Cheng, Stephanie C. Casey, Ryan Case, Melissa Yang, Ray Low, Mark Daris, Bin Fan, Neeraj J. Agrawal, Khaled Ali

**Affiliations:** ^1^Departments of Oncology Research, Amgen Research, South San Francisco, CA, United States; ^2^Pharmacokinetics and Drug Metabolism, Amgen Research, South San Francisco, CA, United States; ^3^Discovery Attribute Sciences, Amgen Research, South San Francisco, CA, United States; ^4^Biologics Discovery, Amgen Research, Thousand Oaks, CA, United States

**Keywords:** cancer, engineered cytokine, IL-21, PD-1, bifunctional fusion, immunotherapy

## Abstract

Inhibitors that block the programmed cell death-1 (PD-1) pathway can potentiate endogenous antitumor immunity and have markedly improved cancer survival rates across a broad range of indications. However, these treatments work for only a minority of patients. The efficacy of anti-PD-1 inhibitors may be extended by cytokines, however, the incorporation of cytokines into therapeutic regimens has significant challenges. In their natural form when administered as recombinant proteins, cytokine treatments are often associated with low response rates. Most cytokines have a short half-life which limits their exposure and efficacy. In addition, cytokines can activate counterregulatory pathways, in the case of immune-potentiating cytokines this can lead to immune suppression and thereby diminish their potential efficacy. Improving the drug-like properties of natural cytokines using protein engineering can yield synthetic cytokines with improved bioavailability and tissue targeting, allowing for enhanced efficacy and reduced off-target effects. Using structure guided engineering we have designed a novel class of antibody-cytokine fusion proteins consisting of a PD-1 targeting antibody fused together with an interleukin-21 (IL-21) cytokine *mutein*. Our bifunctional fusion proteins can block PD-1/programmed death-ligand 1 (PD-L1) interaction whilst simultaneously delivering IL-21 cytokine to PD-1 expressing T cells. Targeted delivery of IL-21 can improve T cell function in a manner that is superior to anti-PD-1 monotherapy. Fusion of engineered IL-21 variants to anti-PD1 antibodies can improve the drug-like properties of IL-21 cytokine leading to improved cytokine serum half-life allowing for less frequent dosing. In addition, we show that targeted delivery of IL-21 can minimize any potential detrimental effect on local antigen-presenting cells. A highly attenuated IL-21 *mutein* variant (R9E:R76A) fused to a PD-1 antibody provides protection in a humanized mouse model of cancer that is refractory to anti-PD-1 monotherapy. Collectively, our preclinical data demonstrate that this approach may improve upon and extend the utility of anti-PD-1 therapeutics currently in the clinic.

## Introduction

Antibodies, that block T cell inhibitory receptors support superior priming and allow dysfunctional T cells to reengage and eradicate established cancers, have transformed the treatment of cancer (1, 2). Despite the success of co-inhibitory receptor antagonists these treatments work for only a small subset of patients (3). PD-1 is a cell surface co-inhibitory receptor expressed on activated T cells (1, 2, 4, 5). When engaged, PD-1 works to constrain T cell function by increasing the threshold for activation leading to diminished anti-tumor immune responses (1, 2, 4, 5). Combinatorial approaches to immunotherapy that use two or more monotherapies can significantly extend the utility of immunotherapies in the clinic (3, 6–9). Specific combinations of cytokine and co-inhibitory receptor agonists or antagonists have proven particularly efficacious in preclinical models of cancer and are now being tested in human trials (8, 10–15). However, this approach remains challenging because of the risks of exacerbated toxicity and the need for complex clinical trial design (6, 7). For cytokine-based therapies numerous challenges exist including pharmacokinetic barriers and immunogenicity, there is also the potential for the activation of inhibitory feedback pathways that can lead to immune suppression, all of which requires careful consideration (16–18).

Interleukin-21 is a type I cytokine and a member of the common cytokine receptor gamma-chain (cg-chain) family that has emerged as a promising immune therapeutic for the treatment of cancer (8). IL-21 that is produced by activated CD4+ T cells and natural killer T (NKT) cells signals via a heterodimeric receptor complex comprised of a discrete IL-21 receptor (IL-21R) subunit together with the cg-chain (19). Activation of the IL-21R complex leads to the activation of the JAK/STAT signaling pathway (20). IL-21R is broadly expressed in hematopoietic cells including T and B lymphocytes, natural killer (NK) cells and myeloid cells (20). Although not an essential growth or differentiation factor, IL-21 is a potent mitogen and survival factor for both NK cells and activated T cells (19, 20). IL-21 can support the differentiation of CD4 + T helper 17 (Th17) as well as follicular helper T cells (Tfh) and can antagonize regulatory T cell (Treg) differentiation. Additionally IL-21 augments the survival of CD8+ T cells resulting in a less activated but more persistent T cell phenotype that leads to enhanced tumor and viral control (8, 19–25). In B cells, IL-21 induces proliferation or apoptosis in a contextual manner and is involved in class switch recombination and optimal plasma cell differentiation (19, 20). A challenging facet of cytokine immunotherapy is that while activating immune cells to potentiate immune responses, the same cytokine can also activate counter-regulatory pathways as exemplified by IL-2 and IFNγ. These counter-regulatory pathways activate protective immune responses, regulatory T cell responses and inhibitory pathways such as PD-L1 (18, 26–32). In dendritic cells (DCs), IL-21 inhibits both maturation and activation and can induce the apoptosis of conventional DCs and in mixed cultures, can potently inhibit the priming of T cells, and may play a role in the induction of tolerance (17, 19, 20).

In humans, IL-21 has been tested as a non-targeted free cytokine in several cancer indications, but despite the promising preclinical data and early phase I clinical data, development of this approach has not progressed further than phase II testing (33, 34). More recently in preclinical models, combination of recombinant IL-21 cytokine together with co-inhibitory receptor antagonists, namely anti-CTLA4 and anti-PD-1 have demonstrated that IL-21 can extend the efficacy of these treatments, and these combinations are now being tested in the clinic (35). However, given the challenges of using cytokines as immunotherapies, it is possible that the preclinical efficacy observed with such combinations may not translate into the clinic. For the reasons discussed above, we hypothesized that to harness the immune potentiating activity of IL-21 it may be prerequisite to address the liabilities of this cytokine, including short half-life and off-target immune suppression. Toward this goal we devised a strategy focusing on an immunocytokine approach that would allow for the delivery of an engineered IL-21 cytokine, in a targeted manner that would circumvent potential liabilities, thus enabling improved exposures and maximizing efficacy.

## Materials and Methods

### Protein Production

The recombinant fusion molecules were produced using a process similar to the process as described by Shi S. Y. et al. (36). Briefly, these molecules were cloned into a pTT5 expression vector and transiently transfected into HEK293-6e suspension cells. Conditioned medium was harvested 6 days post-transfection by centrifugation and then the molecules were purified from conditioned medium using MabSelect SuRe (GE Healthcare) and SP (GE Healthcare) cation exchange chromatography, before formulated into 10 mM acetic acid, 9% sucrose, pH 5.2.

### Human, Cynomolgus Monkey and Mouse PD-1/IL21R Binding Affinity Characterization

IL21R and PD-1 binding affinity were quantitated with ForteBio Octet RED384 and Octet HTX instruments using 384 well plates at 27°C. Unless noted, Octet sample buffer was used for all sample dilution, baselines, association and dissociation steps (10 mM Tris, pH 7.5, 150 mM NaCl, 1 mM CaCl_2_, 0.10 mg.ml BSA, 0.13% (v/v) Triton X-100).

#### IL21R Binding Affinity

Both monovalent IL21R-FLAG-His and bivalent IL21R-Fc recombinant reagents were tested but produced very similar results (within ∼2–3 fold). Human IL21R(1-232)-FLAG-His, cyno IL21R(1-232)-FLAG-His and mouse IL21R(1-236)-FLAG-His were minimally biotinylated (∼1–2 bn/mol) and captured on Streptavidin SAX biosensor tips to a 2.0 nm loading level. The biosensor tips were then incubated in wells containing the anti-PD-1 antibody x IL21 samples in a 3-fold serial dilution. For wildtype IL21 cytokine fusions, the top antibody fusion sample concentration was 10 nM, while for IL21 cytokine mutein fusions the top antibody fusion concentration was 300 nM. An association time of 20 min and a dissociation time of 1.5 h was used to maximize curvature in the active binding sensorgrams for more accurate kinetic fits.

#### PD-1 Binding Affinity

The anti-PD-1 × IL21 antibody fusions were immobilized on amine reactive AR2G biosensor tips through EDC-NHS activation (600 s) followed by immobilization (15 – 20 nM proteins at pH 6 for 2000 s) and then quenched (1 M Ethanolamine, 300 s). After immobilization, the biosensor tips were incubated in Octet running buffer for 300 s (baseline). The final immobilization level for the anti-PD-1 × IL21 antibody fusions was at least 2 nm. The immobilized biosensor tips were then incubated in wells containing a 3-fold serial dilution of the soluble, recombinant PD-1 receptors for human PD-1 (1-170)-FLAG-His, cynomolgus PD-1 (1-167)-FLAG-His or mouse PD-1(25-167)-His (R&D Systems catalog #9047-PD). In all cases, the top PD-1 concentration was 30 nM. Association for 300 s and dissociation for 500 s were used since they empirically produced enough curvature for accurate kinetic fits.

All ForteBio Octet raw data was processed using the ForteBio Data Analysis software v9, v10, or v11: (a) two reference tip curves which had immobilized target but no interaction (i.e., Octet buffer only) were averaged and subtracted from the remaining sample tips curves in the same column; (b) the association and dissociation curves were isolated and aligned to the *Y* axis; (c) the association and dissociation interstep were aligned; (d) Savitzky-Golay filtering was implemented to reduce the high-frequency noise and (e) the resulting set of association and dissociation curves for each sample-target interaction were globally fit with a 1:1 binding model to determine the measured values of the association rate constant *k*_*a*_ (units M^−1^ sec^−1^) and the dissociation rates constants *k*_*d*_ (unit sec^−1^); the equilibrium dissociation constant *K*_*D*_ (units M) was calculated as a ration of the dissociation and association rates constants (=*k*_*d*_/*k*_*a*_).

### Subcutaneous CT26 Tumor Model

Eight-week-old female BALB/c mice (Charles River Laboratories, Hollister, CA, United States) were injected subcutaneously on the right hind flank with 3 × 10^5^ CT26 cancer cells (CRL-2639, ATCC) in 0.1 mL of RPMI media on study day 0. On day 12, tumor volumes were determined, mice were randomized into study groups of ten animals per group, and treatments were initiated: IgG1 isotype 300 μg intraperitoneal (IP) Q3Dx3, anti-PD-1 300 μg IP Q3Dx3, rmIL-21 50 μg IP 3x weekly for 3 weeks, or a combination of rmIL-21 and anti-PD-1. Tumor volumes were measured twice per week. All experimental studies were conducted under protocols approved by the Institutional Animal Care and Use Committee of Amgen (IACUC). Animals were housed at Association for Assessment and Accreditation of Laboratory Animal Care (AAALAC) International-accredited facilities (at Amgen) in ventilated micro-isolator housing on corncob bedding. Animals had access *ad libitum* to sterile pelleted food and reverse osmosis-purified water and were maintained on a 12:12 h light:dark cycle with access to environmental enrichment opportunities.

### Humanized Mouse Model Reconstituted With Human CTLs

NOD.Cg-Prkdc^*scid*^ Il2rg^TM^^1^^*Wjl*^/SzJ (Jax stock number 005557) were used at 6–8 weeks of age. On day 0, animals were reconstituted with 2.5 × 10^6^ freshly thawed CTLs in 100 μl in PBS by retro-orbital injection, 2 × 10^5^ EU IL-2 (Peprotech, catalog # 200-02-1 mg, lot# 11172) in 0.02% BSA in PBS in 100 μl by intraperitoneal injection, and 1 × 10^6^ CMV peptide-expressing luciferase-labeled SKMEL-30 melanoma cells (CMV-SKMEL30-Luc) tumor cells in 100 μl in a 50:50 mixture of growth factor reduced Matrigel (Corning) and serum-free RPMI subcutaneously on the right hind flank. CMV-SKMEL30-luc cells were transduced with the CMV antigens pp65, IE1, and UL138 by lentiviral transduction and blasticidin resistance was used as a selection marker (lentivirus was generated by Applied Biological Materials). The cell line was then luciferase labeled using lentivirus and puromycin selection, MAP tested (IDEXX), and expanded for use *in vivo*. IL-2 was reconstituted according to manufacturer’s protocol. Animals received two additional boosters of IL-2 on d2 and d11. On day 17, tumor volumes were determined, mice were randomized into study groups of ten animals per group, and treatments were initiated: Isotype 300 μg IP Q3Dx3 (BioXCell), anti-PD-1 mAb3 (chimera consisting of anti-human PD-1 variable region and mouse IgG1 constant region) 300 μg IP Q3Dx3, anti-PD-1 mAb3 x R9E:R76A (chimera consisting of anti-human PD-1 variable region, a mouse IgG1 constant region and a C-terminus fusion of human IL-21 variant R9E:R76A) fusion protein monomer 363 μg IP Q3DX3. Tumor volumes were measured twice/week. All experimental studies were conducted under protocols approved by the Institutional Animal Care and Use Committee of Amgen. Animals were housed at Association for Assessment and Accreditation of Laboratory Animal Care International-accredited facilities (at Amgen) in ventilated microisolator housing on corncob bedding. Animals had access *ad libitum* to sterile pelleted food and reverse osmosis-purified water and were maintained on a 12:12 h light:dark cycle with access to environmental enrichment opportunities.

### Cynomolgus Monkey Studies

Experimentally naïve cynomolgus monkeys, 2 to 5 years of age, and weighing 2.7 to 5.7 kg at the onset of the study, were assigned to dosing groups. Blood samples were drawn for pharmacokinetic analysis prior to the first dose and at 0.083, 0.25, 1, 24, 72, 120, 168, 240, and 336 h after a single dose. Serum was separated from blood samples and stored frozen at -80°C and the resulting cell pellet underwent red cell lysis. Serum samples were analyzed for intact drug and the following pharmacokinetic parameters were evaluated from the serum samples: the terminal half-life calculated from the terminal slope of the log concentration-time curve (t_1/2_), maximum concentration (C_*max*_), the time of peak plasma concentration (T_*max*_), and area under the curve (AUC).

Cynomologus monkey studies were conducted under protocols approved by the Charles River Laboratories IACUC. Animals were housed at AAALAC-accredited facilities (Reno, Nevada).

### *In vitro* STAT3 Phosphorylation

HuT78 (ATCC, TIB-161) and HuT78 PD-1 stable cell lines are serum starved for 16 h. HuT78 parental and HuT78 PD-1 stable cell lines (transduced with human PD-1) were then seeded onto separate plates at 40,000 cells per well in the presence of serially diluted antibodies in triplicate for 40 min at 37°C., 5% CO^2^. pSTAT3 Tyr705 levels were measured using AlphaLISA Surefire Ultra pSTAT3 (Tyr705) Assay Kit (Perkin Elmer, #ALSU-PST3-A10K).

### PD-1 Reporter Assay

GloResponse Jurkat NFAT-*luc2/*PD-1 stable effector cells (Promega, #CS187102) and the CHO PD-L1 stable cell line (Promega, #CS178103) were co-cultured at a ratio of 1.25:1 in the presence of serially diluted antibodies in triplicate for 6 h at 37°C., 5% CO^2^. Luminescence was measured using Bio-Glo Luciferase Assay System (Promega, #G7940).

### Mixed Lymphocyte Culture

Mismatched donor pair leukopaks were obtained from AllCells Inc., Donor’s T cells were isolated using Pan T-cell Isolation Kit (Miltenyi Biotec, # 130-096-535) and a mismatched donor’s monocytes were isolated using Pan Monocyte Isolation Kit (Miltenyi Biotec, #130-096-537). Monocytes were further matured for 10 days using CellXVivo Human Monocyte-Derived Dendritic Cell Differentiation Kit (R&D Systems, #CDK004). Pan-T cells were co-cultured with matured monocytes at a ratio of 10:1 in the presence of serially diluted antibodies in triplicate for 72 h at 37°C., 5% CO^2^. Supernatant IL-2 levels were measured by ELISA (Mesoscale Discoveries, #K151QQD-4).

### *In vitro* B Cell Stimulation

Frozen human peripheral blood mononuclear cells (PBMCs) from normal donors were obtained from AllCells, Inc. (Alameda, CA, United States). Frozen cynomolgus PBMCs were obtained from SNBL USA, Ltd. (Everett, WA, United States). To assess the phosphorylation of STAT3 in a mixed human or cynomolgus cell population in response to anti-PD-1-IL21 treatment, frozen human or cynomolgus PBMCs were gently thawed, washed and resuspended with HBSS buffer. Cells were plated onto 96-well round-bottom polypropylene plates at 3–5 × 10^5^ cells/well and treated with various doses of anti-PD-1-IL21 or appropriate controls for 10 min at 37°C, 5% CO_2_. Cells were then washed with cold staining buffer (PBS + 2% FBS) and labeled with Alexa Fluor 488-conjugated mouse αCD3 (SP34-2) (BD Biosciences #557705) followed by a fixable live-dead stain in accordance with the manufacturer’s recommended protocol. Intracellular staining was achieved by fixing the cells with 200 μl of 1X Lyse/Fix Buffer (BD Bioscience #558049) per well for 10 min at 37°C, washing the cells twice with staining buffer, then permeabilizing with 200 μl of cold Perm III Buffer (BD Bioscience #558050) for 30 min on ice. After washing with staining buffer, the cells were stained with PE-conjugated mouse αStat3 (pY705) (BD Bioscience #612569). Cells were then washed twice with staining buffer and then analyzed by flow cytometry.

### *In vitro* Cytotoxic T Cell Assay

#### Expansion of Cytomegalovirus (CMV) Antigen-Specific Cytotoxic T Lymphocytes (CTLs)

Cytomegalovirus antigen-specific CTLs were isolated from PBMCs of CMV seropositive donors. Monocytes were enriched (EasySep Human monocyte isolation kit, Stem Cell Technologies) from the donors and differentiated into dendritic cells (DCs) using the Human Dendritic Cell Differentiation Kit (R&D Systems). The DCs were then matured in the presence of TNF-alpha (R&D Systems), IL-6 (R&D Systems), IL-1 beta (Peprotech), Prostaglandin E2 (Acros organics) and 5 μg/ml pp65 CMV peptide (AnaSpec). Mature DCs were co-cultured with autologous PBMCs in G-Rex flasks (Wilson Wolf) at a ratio of 10:1 PBMC to DC in RPMI + 10% heat-inactivated FBS (Gibco) + 1X sodium pyruvate (Gibco) + 1X non-essential amino acids (Gibco) + 1X β-mercaptoethanol (Gibco). For some experiments cell were primed with 100 nM PD-1 mAb (Amgen) or 100 nM PD-1 X R9E:R76A monomer (Amgen) on day 2 post coculture or left untreated. To determine antigen specificity of the CTLs following expansion, cells were stained with iTAg Tetramer/PE– CMV pp65 tetramer (MBL) 5 days post priming and analyzed by flow cytometry.

#### FACS Characterization of CTLs

Seven days post co-culture, cells were collected, washed and counted. Single-cell suspensions were then stained with fluorochrome-conjugated antibodies and immunofluorescence was analyzed on a FACS Symphony (BD Biosciences) using standard techniques. Antibodies used in this experiment were: anti-CD3(clone:SK7, BD Biosciences) anti-CD8(clone:SK1, BD Biosciences); anti-CD28 (clone:CD28.2, BD Biosciences); anti-CD62L (clone: DREG56, Biolegend); anti-Ki67 (clone:B56, BD Biosciences); anti-CXCR5(clone:RF8B2, BD Biosciences) and anti-PD-1(Amgen).

#### CTL Killing Assay and IFN-Gamma Expression

Nine days post coculture, CD8(+) T cells were enriched from the PBMC: DC cultures and CMV specific CTLs were FACS sorted using standard protocol. Sorted cells were resuspended in RPMI + 5% heat-inactivated FBS (Gibco) + 1X sodium pyruvate (Gibco) + 1X non-essential amino acids (Gibco) + 1X β-mercaptoethanol (Gibco) and rested overnight. The cells were then added into 96-well black-wall clear-bottom plates (Corning) containing pp65 CMV peptide-pulsed luciferase-labeled SKMEL-30 melanoma cells at an effector to target ratio of 2:1. After a 36-h incubation, specific lysis was assessed by adding Bio-Glo reagent (Promega) and reading the plates on the BioTek Synergy Neo2 plate reader (BioTek instruments) using standard luminescence. The supernatants from the above cultures were collected, and IFN-gamma levels were assessed according to manufacturer’s protocol (Meso Scale discovery). In brief, dilution series of controls (detection limit 20,000 pg/mL) and cell culture supernatant (25 μl per well) were transferred to pre-blocked (with 1%w/v solution of Blocker B in PBS) IFN-gamma capture antibody-coated plates and incubated for 2 h at RT, followed by addition of IFN-gamma detection antibody and further incubation of 2 h at RT. The plates were then washed thrice with PBS-0.05% Tween and after addition of read buffer T, the plates were read using a MESO SECTOR S600 (Meso Scale Discovery).

#### Alternative CTL Killing Assay

1 × 10^6 CMV-specific CTLs were washed and resuspended in X-VIVO 15 media (Lonza) then plated in 24-well TC-treated plates (Corning) that have been coated with 0.5 μg/ml anti-CD3 (BioLegend) and 2.5 μg/ml anti-CD28 (BioLegend). Test molecules were added at a final concentration of 500 nM along with 10 U/ml IL-2 (R&D Systems). Following 7 days of incubation at 37°C/5% CO_2_, CTLs were washed, resuspended in RPMI + 10% heat-inactivated FBS (Gibco) + 1X sodium pyruvate (Gibco) + 1X non-essential amino acids (Gibco) + 1X β-mercaptoethanol (Gibco), then titrated into 96-well black-wall clear-bottom plates (Corning) containing pp65 CMV peptide-pulsed luciferase-labeled SKMEL-30 melanoma cells beginning at an effector to target ratio of 20:1. After a 3 day incubation, specific lysis was assessed by adding Bio-Glo reagent (Promega) and reading the plates on the EnVision (PerkinElmer) using standard luminescence settings.

### Statistical Analysis

Graphs were plotted, and statistical significance was established using GraphPad Prism version 7.04 (GraphPad Software, San Diego, CA, United States)^1^. For correlation analysis Pearson correlation co-efficient analysis was used. For comparison of survival curves log-rank (Mantel–Cox) test was used. A Non-linear curve fitting was done using variable slopes (four parameters) method on log-transformed data to establish half maximal effective concentration (EC50) values. Anova with a Tukey’s multiple comparison test was used to calculate statistical differences between groups *in vitro* studies and to compare tumor volumes between the treatment groups. *p* < 0.05 (^∗^) taken as statistical significance (^∗∗^*p* < 0.01, ^∗∗∗^*p* < 0.001, NS, non-significant).

## Results

### Design of Anti-PD-1 and IL-21 Cytokine Fusion Proteins

Recombinant free IL-21 provides modest protection in various preclinical cancer models that is further amplified upon combination with other immune therapies (19, 35, 37). Using a subcutaneous mouse model of colon cancer, we confirmed a combination of recombinant free IL-21 and anti-PD-1 antibody (mAb) dosed concurrently, extended survival, in an established tumor model (Supplementary Figure S1A). IL-21R is expressed broadly throughout the hematopoietic system which significantly impacts cytokine biodistribution and the half-life. Cytokines can be engineered to improve pharmacokinetic properties and therapeutic index; however, most engineered cytokines have only modest improvements in pharmacokinetic properties and often still manifest dose-limiting toxicity and therefore remain constrained to dosing regimens below that of antibodies (15, 30, 38, 39). Moreover, in fusion proteins, the high affinity interaction between the cytokine and its cognate receptor can skew biodistribution away from the targeting antibody noted in previous studies (40). We assessed whether IL-21 could be targeted to PD-1-positive cells by generating antibody cytokine fusion proteins (anti-PD-1 mAb × IL-21) using an anti-PD-1 antibody and the unmodified IL-21 sequence. We avoided fusing the IL-21 cytokine to the N-terminus of the antibody heavy chain or the light chain since this could impact antibody binding to PD-1. We therefore decided to fuse IL-21 to the C-terminus of the antibody heavy chain to preserve bivalency and for optimal targeting. The lysine residue at the C-terminus of the antibody heavy chain was deleted to remediate any potential clipping (41). As depicted in Supplementary Figure S2A, we explored two different designs where the N-terminus of IL-21 was fused to the C-terminus of the antibody heavy chain either with or without (a glycine and serine) linker (GGGGS). In all cases the antibody Fc region was engineered to be devoid of interactions with FcgRs and C1q (SEFL2-2, Supplementary Figure S2A) (42). We confirmed that homodimer fusion proteins, both G4S-linker and linker free variants, could be expressed and we next proceeded to test the cell potency of the fusion molecules. For this we used an IL-21R expressing human T cell line (Hut78) or a variant of this cell line engineered to express PD-1 protein [Hut78 PD-1(+)]. Cells were stimulated with test articles and STAT3 transcription factor phosphorylation was monitored as a surrogate measure of IL-21 pathway activation. As expected, strong phosphorylation of STAT3 was observed in both Hut78 cell lines irrespective of PD-1 expression when they were stimulated with recombinant free WT IL-21 (Supplementary Figure S2B). For the fusion proteins, we observed mild but significant loss in potency and efficacy of STAT3 phosphorylation in the absence of PD-1 expression in the Hut78 parental cells (Supplementary Figure S2B). In contrast, in cells engineered to express cell surface PD-1, we observed complete restoration of STAT3 signaling with evidence for a mild improvement in potency as compared to WT free cytokine (Supplementary Figure S2B). From these results, we determined that fusion of IL-21 to the C-terminus of an antibody can serve to partially attenuate cytokine activity in manner that can be restored by antibody mediated targeting of cell surface PD-1 antigen (Supplementary Figure S2B) (43). Antibody cytokine fusion proteins are known to have altered pharmacokinetic (PK) properties as compared to monoclonal antibodies or recombinant free cytokines. To understand how fusion of IL-21 cytokine to a mAb domain can alter pharmacokinetic properties *in vivo*, we next examined PK properties of a fusion protein consisting of an anti-PD-1 mAb and WT IL-21 (anti-PD-1 × IL-21 WT, homodimer). Anti-PD-1 × IL-21 WT or mAb domain was dosed intravenously into cynomolgus monkeys (Supplementary Figure S3A). The results as shown in Supplementary Figure S3B, demonstrate that as compared to the parent anti-PD-1 mAb the fusion protein has significantly lower exposures and shortened half-life. We thus hypothesized the abundance of IL-21R positive cells, expressed broadly on hematopoietic cells, coupled with the high affinity of the cytokine domain for its cognate receptor is likely to be the primary determinant of biodistribution properties of the fusion protein.

### Design and Characterization of Single Amino Acid Substitution IL-21 Variants

**FIGURE 1:**
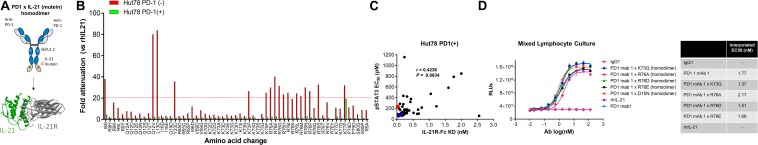
Anti-PD-1 × IL-21 fusion proteins with attenuated IL-21 activity. **(A)** Schematic representation of fusion protein domain assembly. **(B)** IL-21 activity of fusion proteins (tested at 3.7 nM) monitored by STAT3 phosphorylation using AlphaLISA in variants of Hut78 T cells either parental PD-1 (–) or engineering PD-1 (+) cells. Activity of muteins expressed relative to WT recombinant human IL-21 as a measure of attenuation. **(C)** Correlation between potency monitored using STAT3 phosphorylation in PD-1 (+) Hut78 T cells (monitored by AlphaLISA) and binding (determined by Octet) to human IL-21R. *P*-value for ***c*** Pearson correlation co-efficient, *P* = 0.0034 and *r* = 0.4236. **(D)** Potency of single amino acid substitution IL-21 variants fused to anti-PD-1 mAb as homodimer and parental mAb in blocking PD-1/L1 interaction monitored using Promega PD-1/L1 bioassay.

To restrict cytokine activity to targeted cells and thereby further improve PK properties and therapeutic index, we decided to implement a strategy in which the affinity of the IL-21 cytokine for IL-21R was attenuated, our strategy is outlined in Supplementary Figure S4. It is expected that under these conditions cytokine activity can only be delivered in *cis* upon a stabilized interaction between cytokine and cognate receptor, which is enabled by binding of the antibody domain to the targeted cell surface protein. We next proceeded to generate a panel of IL-21 muteins fused to an anti-PD-1 mAb using the linker free homodimer format. Structure guided engineering was used to create a panel of 101 *muteins* each having a single amino acid substitution in the IL-21 amino acid sequence (Table 1). We focused on key amino acid residues in IL-21 that are conserved across (human and cynomolgus monkey) species and mediate the interaction between IL-21 and IL-21R. For the identification of residues that could be mutated to attenuate IL-21 binding to the IL-21R, we utilized the published co-crystal structure of the IL-21: IL-21R complex (PDB ID: 3TGX) (44). Residues within the IL-21: IL-21R were identified and selected for *in silico* mutagenesis to generate a panel of muteins in which each of the selected residues was changed to one of sixteen alternate amino acid residues (except cysteine, phenylalanine and tryptophan), using MODELER tool (Biovia Discovery Studio) to optimize conformation. In order to quantify the probable impact of each mutation on the binding of IL-21 to IL-21R, ΔΔG_*mut*_ (where ΔΔG_*mut*_ is the difference between the calculated binding free energy, ΔΔG_*bind*_, of the mutated structure and the wild type structure and ΔΔG_*bind*_ is the difference between the free energy of the complex and the unbound proteins) was calculated by using the Biovia Discovery Studio software (45). Mutations that led to ΔΔG_*mut*_ >1 kcal/mol were selected for further analysis. Further residues for mutation were also identified by visual inspection of the IL-21: IL-21R complex structure (PDB ID: 3TGX) and the unbound structure (PDB ID: 2OQP) of IL-21 (44). Additional residues were selected in region 56-83 (residues R65, I66, V69, S70, K72, K73, K75, R76, K77, and S80) of IL-21 which has previously been reported to exhibit partial helix and disorder forms, and is present in the IL-21R binding interface (46). Each of the selected residues within region 56–83 were mutated to glycine and proline residues with the goal of disrupting the helix structure of this region to disfavor the bound conformation of the IL-21 (Table 1). Biophysical and functional properties of the fusion proteins were determined, and for the IL-21 domain these attributes were compared to those of the WT free cytokine (Table 2 and Figure 1). Equilibrium dissociation constant (K_*D*_) was determined for IL-21R for free WT IL-21 and for each of the fusion proteins (Table 2). Since mutations in the IL-21 receptor binding domain impinge on the affinity of the cytokine for IL-21R, it was not possible to assign an accurate K_*D*_ in many of the muteins (Table 2).

**TABLE 1:** IL-21 residues selected for substitution.

	IL-21 aa																		

Position	I16	I66	I8	K72	K73	K75	K77	L13	P78	Q12	Q19	R5	R65	R76	R9	S70	S80	V69	Y23
Substituion	I16D	I66D	I8A	K72D	K73A	K75D	K77D	L13D	P78D	Q12A	Q19D	R5A	R65D	R76A	R9A	S70E	S80G	V69D	Y23D
	I16E	I66G	I8D	K72G	K73D	K75G	K77G		P79D	Q12D		R5D	R65G	R76D	R9D	S70G	S80P	V69G	
		I66P	I8E	K72P	K73E	K75P	K77P			Q12E		R5E	R65P	R76E	R9E	S70P		V69P	
			I8G		K73G					Q12N		R5G		R76G	R9G	S70Y			
			I8N		K73H					Q12S		R5H		R76H	R9H				
			I8S		K73I					Q12T		R5I		R76I	R9I				
					K73N					Q12V		R5K		R76K	R9K				
					K73P							R5L		R76L	R9L				
					K73Q							R5M		R76M	R9M				
					K73S							R5N		R76N	R9N				
					K73V							R5Q		R76P	R9Q				
												R5S		R76Q	R9S				
												R5T		R76S	R9T				
												R5V		R76T	R9V				
												R5Y		R76V	R9Y				
														R76Y					

**TABLE 2:** IL-21 *mutein* binding to human IL-21R.

	IL21R-Fc		IL21R-Fc

Variant	KD (nM)	Variant	KD (nM)
rhlL-21	0.027	V69D	0.040
Anti-PD-1 mab 1 x IL-21 WT	0.079	V69G	0.21
R5D	No binding	V69P	2.0
R5E	No binding	S70E	0.95
R5G	No binding	S70G	0.52
R5G	Weak binding	S70P	**∼**10
R5I	Weak binding	K72D	0.24
R5K	Weak binding	K72G	0.25
R5L	Weak binding	K72P	9.0
R5M	Weak binding	K73A	0.053
R5N	No binding	K73D	0.44
R5Q	2.100	K73E	0.073
R5S	Weak binding	K73G	0.25
R5T	Weak binding	K73H	0.19
R5V	Weak binding	K73I	0.17
R5Y	Weak binding	K73N	0.074
I8A	Weak binding	K73P	**∼**2
I8D	Weak binding	K73Q	0.069
I8E	No binding	K73S	0.17
I8G	weak binding	K73V	1.1
I8N	Weak binding	K75D	20
I8S	**−**4	K75G	0.16
R9A	6.836	K75P	**∼**1
R9D	>100	R76A	**∼**11
R9E	No binding	R76D	**∼**12
R9G	**−**40	R76E	18
R9H	0.084	R76G	**∼**2
R9I	2.2	R76H	**∼**2
R9K	2.0	R76I	0.32
R9L	1.6	R76K	**∼**0.2
R9M	Weak binding	R76L	**∼**0.2
R9N	Weak binding	R76M	**∼**0.6
R9Q	Weak binding	R76N	15
R9S	Weak binding	R76P	**∼**0.4
R9T	Weak binding	R76Q	0.77
R9V	Weak binding	R76S	1.1
R9Y	0.063	R76T	0.11
Q12A	0.23	R76V	1.8
Q12D	0.42	R76Y	0.27
Q12E	0.031	K77G	0.66
Q12N	0.38	K77P	2.1
Q12S	0.32	P78D	1.2
Q12T	<0.26	P79D	0.32
Q12V	<2.2	S80G	0.27
L13D	11	S80P	0.31
I16D	0.094	R5A	0.24
I16E	0.076	S70Y	0.24
Q19D	0.17		
Y23D	1.7		
R65D	0.088		
R65G	0.13		
R65P	0.90		
I66D	0.68		
I66G	2.6		
I66P	7.1		

**FIGURE 2:**
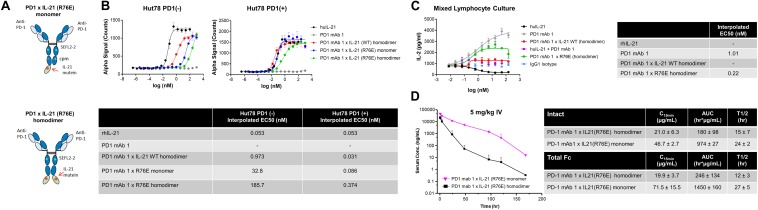
Characterization of IL-21 R76E single substitution variant with attenuated activity. **(A)** Schematic representation of monomer IL-21 (upper panel) and homodimer (lower panel) fusion proteins. **(B)** IL-21 activity of R76E variant or free WT IL-21, monitored using STAT3 phosphorylation (AlphaLISA) in Hut78 PD-1(–) cells (left panel) and engineered Hut78 PD-1 (+) cells (right panel). **(C)** Activity of free WT IL-21, fusion proteins or parental mAb in mixed lymphocyte cultures of alloreactive pan-T cells and dendritic cells. *N* = 2 cynomolgus monkeys/group in **c** dosed 5 mg/kg with single dose of homodimer or monomer IL-21 fusion proteins. **(D)** Mean plasma concentration-time profiles of anti-PD-1 × R76E monomer and anti-PD-1 × R76E homodimer fusion proteins (upper panels), with summary of pharmacokinetic parameters (lower panels).

We also tested the *in vitro* activity of the fusion molecules using our engineered Hut78 cell lines. We report that in agreement with the binding data, because of the high degree of attenuation for IL-21R, we observed attenuated STAT3 phosphorylation in the absence of PD-1 expression in the Hut78 parental cells. In contrast, in cells engineered to express cell surface PD-1, we observed significant restoration of STAT3 signaling, but signaling was still partially attenuated as compared to free wildtype cytokine (Figure 1B). For those muteins where we could measure both affinity and potency in Hut78 PD-1 expressing cells, we were able to confirm a positive correlation between cell activity and the affinity of the molecules (Figure 1C). To test the blocking activity of the PD-1 mAb arm of the fusion protein, we used a reporter gene assay (Promega) in which PD-1-expressing Jurkat effector cells are incubated with antigen presenting cells expressing PD-L1 in the absence or presence of PD-1 blocking antibodies. The results, shown for a subset of the fusion proteins, suggest that the fusion proteins retain the ability to bind and block the PD-1 pathway with similar potency to the parent anti-PD-1 mAb (Figure 1D). For a more detailed characterization of the impact of mutations that disrupt binding of IL-21 to IL-21R, we selected a single anti-PD-1 × IL-21 variant (R76E) which had preferred attributes of high attenuation of activity in PD-1 (−) but retained significant activity in PD-1 expressing cells. In addition, since improving pharmacokinetic properties is important for both sustained blockade of the PD-1 pathway as well providing a more prolonged IL-21 signal, and since it has previously been demonstrated that the valency of Fc-fusion proteins can significantly affect PK properties, we decided to test differences between a homodimer versus a monomeric IL-21 fusion proteins (47). Variant anti-PD-1 x R76E was cloned and expressed with IL-21 domain fused to each heavy chain resulting in a fusion molecule with two IL-21 domains (homodimer), and a configuration where the IL-21 domain was fused to only one of the heavy chains resulting in a fusion molecule with only one IL-21 domain (monomer). In the case of IL-21 monomer, to achieve a heterodimer consisting of a single IL-21 subunit and a bivalent mAb, charge pair mutations (cpm) in the Fc domain were used to drive heterodimeric association of the individual heavy chains of the mAb domain (Figure 2A) (43). As shown in Figure 2B, variant anti-PD-1 × R76E has attenuated IL-21 activity on PD-1 (−) cells which is restored upon PD-1 expression. Compared to a WT IL-21 fusion protein, the anti-PD-1 × R76E (monomer and homodimer) has a more attenuated activity. In addition, we find that the monomer variant of anti-PD-1 × R76E has a modest improvement in potency over the homodimer variant. We next wanted to understand using a more complex *in vitro* system the potential for off-target activation of non-targeted IL-21R expressing cells, as it is known that IL-21 can be immunosuppressive when exposed to antigen presenting cells, and can potently inhibit the alloresponse in mixed lymphocyte cultures (MLC, Figure 2C) (17). As shown in Figure 2C, we confirm that free IL-21 can potently suppress the alloresponse and that IL-21-mediated immune suppression is dominant when free-IL-21 and anti-PD-1 are combined as monotherapies. We also tested WT and anti-PD-1 × R76E variants of IL-21 fusion proteins, and report that in the absence of any attenuation WT IL-21 fusion protein can also potently suppress the alloresponse response. When we examined the behavior of anti-PD-1 × R76E variant, we observe a subtle but significant suppression of the response at higher concentrations. To determine the *in vivo* characteristics of the anti-PD-1 × R76E variants, pharmacokinetic parameters (PK) were determined using cynomolgus monkeys. As shown in Figure 2D, both monomer and homodimer variants exhibit distinct PK profiles, with the monomer showing superior exposures and half-life. To address potential liabilities relating to non-specific signaling that could translate into immune suppression mediated through the action on dendritic cells, and to further explore the potential for using a monomeric format to improve pharmacokinetic properties, we proceeded to generate more attenuated IL-21 variants.

### Design and Characterization of Dual Amino Acid Substitution IL-21 Variants With Reduced Off-Target Signaling

**FIGURE 3:**
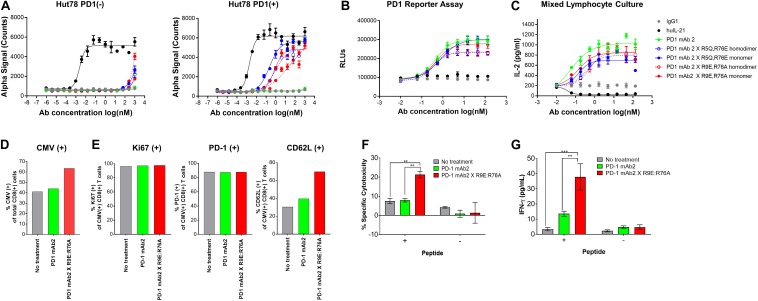
Characterization of IL-21 variants with dual amino acid substitutions. **(A)** Activity of fusion proteins and free WT IL-21 in Hut78 PD-1(–) cells (left panel) and engineered Hut78 PD-1 (+) cells (right panel) monitored using STAT3 phosphorylation (AlphaLISA) as a surrogate measure of IL-21 activity. **(B)** Potency of fusion proteins and parental mAb in blocking PD-1/L1 interaction monitored using Promega PD-1/L1 bioassay. **(C)** Activity of free WT IL-21, dual amino acid substation variants or parental mAb in mixed lymphocyte cultures of alloreactive pan-T cells and dendritic cells. **(D–E)** CTLs derived from PBMCs from Donor 1 under indicated priming conditions. Seven days post co-culture, proportions of **(D)** CMV antigen specific CTLs and **(E)** Ki67 (left-panel), PD-1 (middle-panel) and L-selectin (CD62L, right-panel) was analyzed by FACS analysis. CTLs primed under different conditions were isolated and cocultured with peptide pulsed melanoma cells for 36 h to examine **(F)** cytotoxicity against peptide-loaded melanoma cells, determined by measuring luciferase activity and **(G)** CTL IFN-gamma production. Experiments in **(F)** and **(G)** were conducted in triplicates and the error bars represent SEM. *P*-values were calculated using one-way Anova with a Tukey’s multiple comparison test. *P*-values: *** < 0.001, ** < 0.01.

To further reduce non-specific IL-21 signaling, a second panel of molecules was constructed (Table 3). Using the known IL-21/IL-21R structure to help guide selection, single amino acid substitution variants with the greatest degree of attenuation, as determined using cell and binding assays were combined to create a panel of double mutant variants fused as a monomer or homodimer to the C-terminus heavy chain of a bivalent anti-PD-1 antibody (Table 3 and Supplementary Figure S5A). A subset of the double mutant variants was evaluated for binding to IL-21R (Table 4 and Supplementary Figure S7). Consistent with the greater degree of attenuation, we were unable to establish *K*_*D*_ values for the interaction between fusion protein(s) and IL-21R, and we determined that these values are higher than the top concentration in the assay (300 nM), as such relative attenuation as compared with free WT cytokine is estimated to be >1000 fold for these more attenuated molecules (Table 4 and Supplementary Figure S7). We next tested cell activity assays using a smaller subset of the double mutant constructs (Figure 3 and Supplementary Figures S5b,c). According to our hypothesis, cell association of fusion proteins in which IL-21/IL-21R association has been disrupted can be restored through binding of the mAb domain to a cell surface receptor allowing for the stabilized interaction between IL-21 and IL-21R. In line with our hypothesis, double muteins demonstrate a high degree of attenuation (>1000 fold as compared with free WT IL-21 cytokine) for STAT3 activation in cells devoid of PD-1 expression (Figure 3A and Supplementary Figure S6). Activity can be restored in cells engineered to express cell surface PD-1, but still partially attenuated as compared with free wildtype cytokine (Figure 3A and Table 4). We also confirmed that the fusion proteins consisting of the more attenuated IL-21 variants retain the ability to block the PD-1/PD-L1 interaction (Figure 3B and Table 4). We next proceeded to test if additional attenuation could protect against non-specific activation of bystander IL-21R expressing APCs in a mixed culture system using alloreactive T cells that respond to antigen peptide complexes presented by dendritic cells (Figure 3C and Table 4). In contrast to recombinant WT IL-21 cytokine, which completely suppressed the alloresponse, the fusion proteins have similar activity to the parental anti-PD-1 mAb (Figure 3C). Our data suggest that in the absence of PD-1 expression the more attenuated fusion proteins fail to activate bystander cells expressing IL-21R in these conditions, and in the context of an alloresponse the fusion proteins have only limited signaling in *trans* allowing for the preservation of DC function. We next tested, the impact of PD-1 × IL-21 fusion proteins on the differentiation and effector function of cytotoxic T cells (CTL) derived from PBMCs. For this we tested the activity of PD-1 × IL-21 fusion protein using two CMV seropositive donors across four independent experiments (Figures 3D–G and a second independent donor Supplementary Figure S8). Antigen specific CTLs were generated by co-culturing of peripheral blood mononuclear cells (PBMCs) and autologous peptide-loaded DCs in the presence of a PD-1 × IL-21 fusion protein and for comparison, PD-1 mAb or untreated cell were used as controls. After 7 days of co-culture with DCs, cell surface and intracellular markers of T cell proliferation and activation were monitored on antigen-specific CD8+ T cells. We report that T cell priming in the presence of PD-1 × IL-21 fusion protein gives rise to a mild but reproducible increase in the frequency of antigen-specific CD8+ T cells as compared to untreated control or PD-1 mAb treatment groups (Figure 3D and Supplementary Figure S8A). The increase in the frequency of antigen-specific cells was not correlated to increase in proliferation as the percentage of antigen-specific CD8+ T cells expressing Ki67 was equivalent across all treatment groups. We examined two further cell surface markers, namely PD-1 and L-selectin (CD62L), as markers of T cell activation and differentiation, respectively. Similar proportions of PD-1 positive T cells were observed across all treatment groups and was consistent across independent donors (Figure 3E and Supplementary Figure S8A). We also monitored L-selectin a marker enriched on naïve and memory T cells. Previously it has been reported that IL-21 promotes the acquisition of alternative effector phenotype with increased L-selectin (48). We report that priming of T cells in the presence of PD-1 x IL-21 fusion protein leads to an increase in proportion of L-selectin (CD62L) positive CTLs as compared to PD-1 mAb and untreated treatment groups (Figure 3E and Supplementary Figure S8A). These data suggest that in the presence of PD-1 × IL-21 CTLs can acquire an effector phenotype but retain the naïve marker L-selectin. We next examined effector function of differentiated CTLs by co-culturing CTLs together with peptide-loaded tumor cells. We report that CTLs conditioned with PD-1 × IL-21 fusion protein demonstrated superior cytotoxicity and IFN-gamma production as compared to untreated control or those primed together with a PD-1 mAb (Figures 3F–G and Supplementary Figure S8). We extended our studies to examine the effect of PD-1 × IL-21 fusion proteins on cytotoxicity of differentiated effector cells (Supplementary Figure S9). For these studies, to more faithfully mimic the clinical setting, in which fusion protein is expected to augment pre-existing immune responses, we used *in vitro* differentiated mature CTL lines. These were activated with a combination of CD3/28 beads (to mimic a chronic activation conditions) together with either anti-PD-1 mAb or fusion protein after which the CTLs were co-incubated with peptide pulsed PD-L1 (+) cancer cells. Our data suggest that under these conditions, CTLs treated with fusion protein have superior effector functions including cytotoxicity and IFN-gamma production versus anti-PD-1 mAb (Figures 3D–F and Supplementary Figures S8, S9).

**TABLE 3:** IL-21 residues selected for substitution and generation of double mutants.

Double mutants
R9E, R76E
R9A, R76E
R5E, R76E
R5A, R76E
R5Q, R76E
R9E, R76A
R9A, R76A
R5E, R76A
R5A, R76A
R5Q, R76A

**TABLE 4:** Summary of *in vitro* attributes of anti-PD-1 x IL-21 double *muteins.*

	Hut78 PD-1 (–)	Hut78 PD-1(+)	PD-1 reporter	Mixed lymphocyte				
	Interpolated	Interpolated	Interpolated	culture Interpolated	hulL-21R	cylL-21R	huPD-1	cyPD-1
	EC50 (nm)	EC50 (nm)	EC50 (nm)	EC50 (nm)	KD (nM)*	KD (nM)*	KD (nM)*	KD (nM)*
IgGl	–	–	–	–	–	–	–	–
rhlL-21	0.003	0.002	–	–	0.029	0.044	–	–
PD-1 mAb 2	–	–	0.487	0.161	–	–	0.90	1.33
PD-1 mAb 2 x R5Q:R76E homodimer	>1000	1.1	0.367	0.518	>300	>300	0.68	1.27
PD-1 mAb 2 x R5Q:R76E monomer	>1000	0.28	0.809	0.249	>300	>300	0.56	1.28
PD-1 mAb 2 x R9E:R76A homodimer	>1000	4.42	0.308	0.625	>300	>300	0.71	1.42
PD-1 mAb 2 x R9E:R76A monomer	>1000	0.78	0.503	0.241	>300	>300	0.90	1.61

*^∗^Sensograms are shown in Supplementary Figure S7.*

### *In vivo* Characterization of Dual Amino Acid Substitution IL-21 Variants With Improved Pharmacokinetic Properties and Superior Efficacy *in vivo* in an Anti-PD-1 Refractory Setting

**FIGURE 4:**
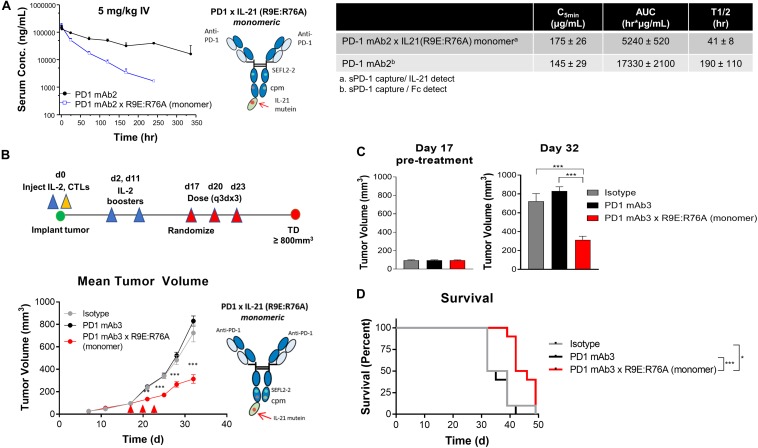
*In vivo* characterization of dual amino-acid substation IL-21 fusion proteins **(A)** Mean plasma concentration-time profiles (upper left panel) of variant anti-PD-1 × R9E:R76A monomer (depicted, upper right and parent anti PD-1 mAb, with summary of pharmacokinetic parameters (lower panels). **(B–D)** *In vivo* activity of fusion protein administered to humanized NOD.Cg-Prkdc^*scid*^ Il2rg^*tm*1*Wjl*^/SzJ mouse model engrafted with human melanoma cell line CMV-SKMEL-30-Luc expressing cytomegalovirus (CMV) antigen and reconstituted with human CMV-reactive CTLs adoptively transferred by retro-orbital injection on day 0, followed by randomization of mice with established (100 mm^3^ tumors) on day 17 (*N* = 10 mice per group) and therapeutic administration of either isotype control mAb, PD-1 mAb3 or PD-1mAb3 × R9E:R76A monomer fusion protein administered by intraperitoneal injections **(B)** Summary of experimental design (upper-panel) and *in vivo* activity as measured by tumor volume (lower-panel). *P*-values were calculated with one-way Anova with Tukey’s *post hoc* test and were as follow; Day 21: *P* = 0.0023 (PD-1 mAb3 vs. PD-1 mAb3 × R9E:R76A monomer) and *P* = 0.0056 (Isotype vs. PD-1 mAb3 × R9E:R76A monomer); Day 24: *P* = 0.0001 (PD-1 mAb3 vs. PD-1 mAb3 × R9E:R76A monomer) and *P* = 0.0001 (Isotype vs. PD-1 mAb3 × R9E:R76A monomer); Day 28: *P* = 0.0001 (PD-1 mAb3 vs. PD-1 mAb3 × R9E:R76A monomer) and *P* = 0.0012 (Isotype vs. PD-1 mAb3 × R9E:R76A monomer); Day 32: *P* = 0.0001 (PD-1 mAb3 vs. PD-1 mAb3 × R9E:R76A monomer) and *P* = 0.0001 (Isotype vs. PD-1 mAb3 × R9E:R76A monomer). Repeated Measures two-way Anova with Tukey’s *post hoc* test for entire curve: Isotype vs. PD-1 mAb3: NS. Isotype vs. PD-1 mAb3 × R9E:R76A monomer: *P* = 0.0001. PD-1 mAb3 vs PD-1 mAb3 × R9E:R76A: *P* = 0.0001. **(C)** Summary of tumor volume at randomization (day 17) and pre-treatment (left panel) and at day 32 (right panel) *P*-values were calculated using one-way Anova with a Tukey’s *post hoc* test. *P* = 0.0001 (PD-1 mAb3 vs. PD-1 mAb3 × R9E:R76A monomer) and *P* = 0.0001 (Isotype vs. PD-1 mAb3 × R9E:R76A monomer). **(D)** Survival analysis of tumor bearing mice. *P*-values of log-rank (Mantel-Cox) test were as follow; *P* = 0.0037 (Isotype vs. PD-1 mAb 3 × R9E:R76A monomer), *P* = 0.0001 (PD-1 mAb 3 monotherapy vs. PD-1 mAb 3 × R9E:R76A monomer). *P*-values: *< 0.05, ***< 0.001.

We next wanted to extend our observations to understand pharmacokinetic properties of the more attenuated dual amino-acid substitution IL-21 fusion proteins. For this we used monomer fusions proteins because of their superior PK properties; groups of animals were dosed with fusion protein or parental mAb and PK parameters were calculated. The results as shown in Figure 4A suggest that attenuated cytokine variants have substantially improved PK properties as compared to first generation anti-PD-1 × R76E *mutein* (Figure 2). We extended our observations to explore *in vivo* activity of our fusion proteins. Since human IL-21 does not cross-react with mouse IL-21R and in the absence of an appropriate mouse surrogate molecule, we decided to implement a humanized mouse system; for this we used humanized mice, which were engrafted with human (PD-L1+) melanoma cells (SKMEL-30-Luc) engineered to express a model antigen (CMV-SKMEL-30-Luc, expressing peptide antigen derived from cytomegalovirus, CMV) and either a human-mouse chimeric PD-1 mAb, with a variable domain recognizing human PD-1 and a constant Fc-region from mouse IgG1, or a fusion protein consisting of the same parent PD-1 mAb and a monomeric variant of human IL-21 R9E:R76A (Figure 4B and Supplementary Table S1). On the same day as tumor engraftment, mice received adoptively transferred antigen (CMV)-specific CTLs, which we confirmed, demonstrate potent *in vitro* cytotoxicity against the antigen-expressing cancer cells (Figure 3F). In this model, the failure of tumor reactive CTLs to control cancer growth leads to development of progressive tumors which are palpable by day 17. Therapeutic administration (into mice with ∼100 mm^3^ established tumors) with an isotype control antibody or an anti-PD-1 mAb failed to resolve the disease or have any discernable impact on tumor growth, establishing this tumor model as both “high bar” and PD-1 refractory (Figures 4B,C). In contrast, therapeutic administration of a PD-1 × IL-21 fusion protein (Supplementary Table S1), has a significant inhibitory effect on the tumor growth and improves overall survival (Figures 4B–D). Collectively our data support the idea that chronic activation of T cells can lead to a diminished anti-tumor immune response, and that administration of a fusion protein consisting of a PD-1-targeted IL-21 moiety can significantly extend the function of CTLs and support superior tumor control in a mouse model that is refractory to PD-1 mAb monotherapy.

## Discussion

Inhibitors of T cell coinhibitory receptors such as anti-PD-1 and anti-CTLA4, can improve antitumor immunity. However, most patients remain refractory to these therapies (6). The effectiveness of coinhibitory receptor antagonists maybe extended in combination with additional modalities, including cytokines that function through complementary mechanisms (6, 7, 15, 29). Cytokines are small proteins that are essential in shaping protective antitumor immune responses, however, the utility of cytokines in the clinic for cancer immunotherapy is limited, with only TNFα, IFNα, and recombinant IL-2 approved for a small number of cancer indications (29, 49, 50). The inclusion of cytokines into therapeutic regimens faces considerable challenges, largely due to dose limiting toxicities and short serum half-life (6, 29). Engineered fusion proteins, where cytokines are genetically fused to an IgG antibody or a fragment thereof, commonly known as immunocytokines, can significantly extend half-life of cytokines, and improve safety by enabling targeted delivery to a specific cell or tissue. In the case of cytokines that present systemic toxicity or those that can both potentiate as well as suppress immune responses, such as IL-2 and IL-21, an immunocytokine approach can serve to harness the potentially beneficial biology whilst limiting any detrimental impact to the host (10, 26, 29, 31, 38, 49). Many possible configurations can be considered when designing immunocytokines that can specify the nature of how the cytokine interacts with its target cell population or the local environment (49). Depending on the desired outcome, cytokines can be enriched in the tumor environment through tumor cell targeting antigens, with the view to (in *trans*) activate infiltrating local immune cells. Alternatively, cytokines can be delivered in *cis* directly to immune cells that are known to be enriched in the tumor environment *via* cell surface receptors expressed on leukocytes (Supplementary Figure S4). Additional considerations when designing cytokine fusion proteins include the nature of the Fc receptor interaction and how the cytokine domain is fused through N or C terminus fusion to IgG heavy or light chain can also significantly influence outcomes such as target cell expansion versus depletion as well as biodistribution and efficacy. The technical challenges of implementing immunocytokines is exemplified by recent examples of IL-2 fusion proteins (40, 43). Fusion of an antibody and cytokine can have undesirable outcomes for both arms of the molecule. High-affinity association of cytokine and its cognate receptor can alter distribution in favor of fast clearance, and in cases where there is a large population of non-targeted, cytokine receptor-expressing immune cells, can lead to increased toxicity (40). In the case of a fusion protein where the antibody domain has a function in addition to acting as a targeting moiety, as in the case of an antagonist antibody such as anti-PD-1, where prolonged blockade of the targeted receptor is needed, this can lead to loss of target coverage and efficacy. Whilst cytokine attenuation can improve toxicity profiles, including acute toxicities, such as cytokine storm, chronic low-level activation of cytokine pathways can still lead to the same undesirable outcome in the longer term. Moreover, to achieve or maintain desirable dosing properties the nature of the attenuating mutations need to be carefully considered, as simply attenuating cytokine activity to remediate undesirable properties such as off-target interactions may not improve, and can even worsen PK properties of the molecule (26, 28, 29).

The IL-21 cytokine has generated considerable interest as a potential immunotherapy, but in addition to the liabilities common to all cytokines including a short-half life, IL-21 can also suppress dendritic cell function and by extension priming of immune responses (8, 19, 26, 33–35, 51, 52). Using a structure guided protein engineering approach, we have combined an engineered IL-21 cytokine domain and a PD-1 blocking antibody into a bifunctional fusion protein. To overcome the limitations of IL-21 cytokine and to improve efficacy, we have used an iterative approach to design IL-21 variants with increasing degrees of attenuation that are masked from binding to IL-21R in the absence of PD-1 receptor co-expression. Our approach allows for delivery of IL-21 as well as sustained PD-1 blockade with molecules that remain stable *in vivo* over prolonged periods. Using *in vitro* assays, we show that priming in the presence of an PD-1 × IL-21 fusion protein leads to enhanced cytotoxicity and effector cytokine production in antigen-specific CD8 + T cells. Moreover, in a mouse model of cancer, we demonstrate that when dosed into an anti-PD-1 mAb refractory tumor model, fusion proteins can engage tumor specific CD8+ cells to provide superior tumor control in a manner which is superior to an antagonist anti-PD-1 mAb monotherapy. Collectively our data demonstrate that this approach can harness orthogonal pathways, by antagonizing the PD-1/L1 inhibitory pathway whilst activating IL-21 cytokine signaling in a targeted manner to augment CD8+ T cell cytolytic effector function.

A significant advantage of our approach is that it allows for concentration of cytokine in a spatially restricted manner and activation of cytokine signaling in a specific population of T cells, namely PD-1 expressing cells. In addition, we show that a highly attenuated IL-21 mutein variant (R9E:R76A) has prolonged exposures and improved serum half-life as compared to recombinant free IL-21 cytokine, allowing for a longer duration between treatment cycles and a more simplified clinical trial design (33). Delivery of IL-21 cytokine to T cells as opposed to other IL-21R expressing cells including myeloid cells can overcome immune suppression associated with activation of STAT3 signaling in these cells (17). In summary, these preclinical data support the testing of these molecules across a wide range of cancer indications, including T cell infiltrated and/or PD-L1 expressing tumors previously refractory to PD-1/L1 inhibitors. Our data suggest a fusion protein approach can overcome the current limitations of these inhibitors and may extend the activity of this class of molecules in previously refractory cancer indications.

## Data Availability Statement

All datasets generated for this study are included in the article/Supplementary Material.

## Ethics Statement

The animal study was reviewed and approved by the Charles River Laboratories IACUC The Institutional Animal Care and Use Committee of Amgen (IACUC).

## Author Contributions

KA conceived and designed the project. NA conducted the computational analysis and designed mutations. JP guided the design pharmacokinetic studies and interpreted the data. SS, GS, DO, AS, and AL performed *in vitro* experiments and interpreted the data. JD guided the design of *in vivo* mouse studies. JC conducted *in vivo* mouse studies and interpreted the data. SC designed and performed *in vivo* mouse studies and interpreted the data. RC designed binding studies and interpreted the data. MY, RL, BF, and MD were involved in the design, cloning, expression, and purification of protein reagents. KA wrote the manuscript, with input from all authors.

## Conflict of Interest

SS, JD, JC, SC, RC, MY, NA, and KA are full-time employees and share-holders of Amgen Inc., JP is an employee of Merck, MD is an employee of A2 therapeutics, GS is an employee of Celgene, BF in an employee of NGM Biopharmaceuticals. DO is an employee of Spotlight Therapeutics. The remaining authors declare that the research was conducted in the absence of any commercial or financial relationships that could be construed as a potential conflict of interest.

## Footnotes

1 http://www.graphpad.com
