# Supplementary material for: Engineered IL-21 Cytokine Muteins Fused to Anti-PD-1 Antibodies Can Improve CD8+ T Cell Function and Anti-tumor Immunity
Source: Front Immunol. 2020 May 8;11:832. doi: 10.3389/fimmu.2020.00832 (PMC7225340; doi:10.3389/fimmu.2020.00832)
Supplement: Supplementary file 1 [file Data_Sheet_1.pdf]

Supplementary Figure 1.

A.

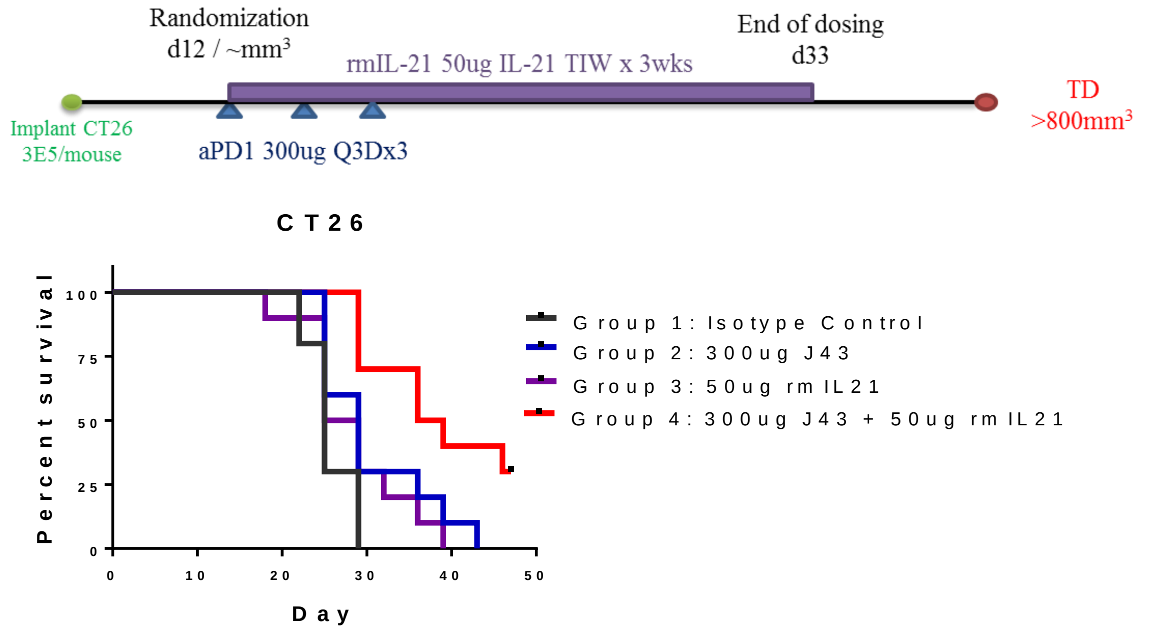

Supplementary Figure 1. *In vivo* activity of a combination of IL-21 and anti-PD-1 mAb and impact of free IL-21 on dendritic cells. (A) Survival analysis of CT26 tumor bearing Balb/c female (*N* =10 per group) mice administered either anti-PD-1 mAb alone, free WT IL-21 or a combination of free WT IL-21 and anti-PD-1 mAb. *N = 10* Balb/C, *P* values of log-rank (Mantel-Cox) test were as follows; *P* = 0.0002 (Isotype IgG1 vs rmIL-21 + anti-PD-1 (J43)), *P* = 0.0408 (Isotype vs anti-PD-1 (J43)), *P* = 0.1269 (Isotype vs rmIL-21), *P* = 0.0143 (anti-PD-1 (J43) vs rmIL-21 + anti-PD-1 (J43)), *P* = 0.0047 (rmIL-21 vs rmIL-21 + anti-PD-1 (J43)). $*P < 0.05$, $**P < 0.01$, $***P < 0.001$.

Supplementary Figure 2.

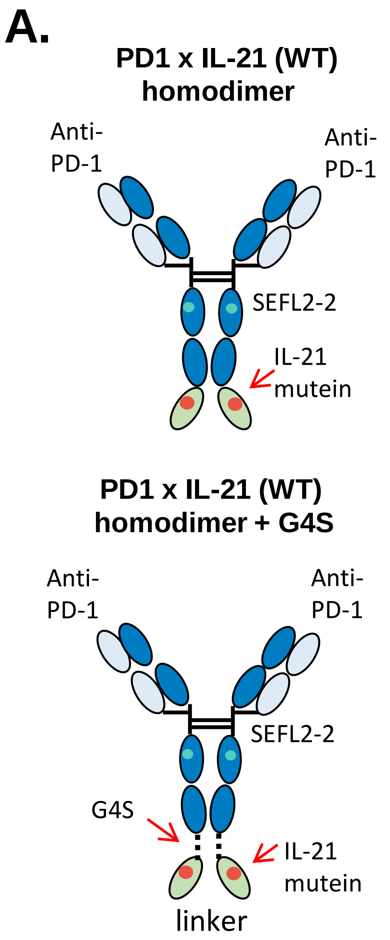

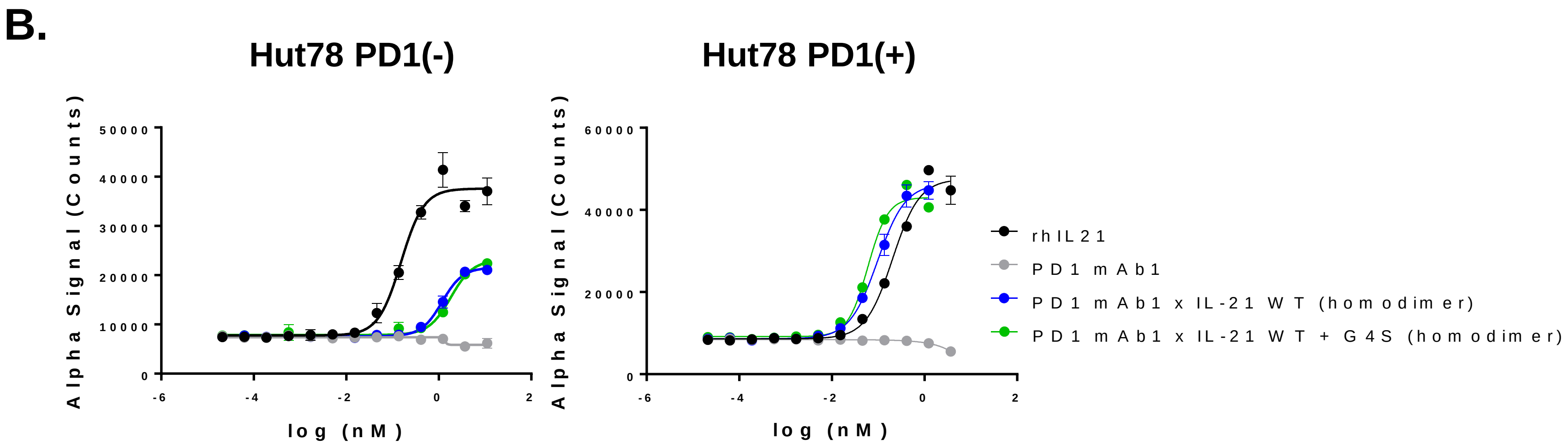

	Interpolated EC50 PD1 (-) (nM)	Interpolated EC50 PD1(+) (nM)
rhIL-21	0.132	0.184
PD1 mAb 1	-	-
PD1 mAb 1 x IL-21 WT homodimer (-) G4S	1.143	0.062
PD1 x mAb 1 x IL-21 WT homodimer (+) G4S	>3.7	0.045

Supplementary Figure 2. Anti-PD-1 x IL-21 bifunctional fusion protein designs and activity. (A) Schematic representation of IL-21 fusion protein designs. (B) Activity of fusion proteins expressed as homodimer or monomer with or without linker and compared to free WT IL-21 in Hut78 PD-1(-) cells (left panel) and engineered Hut78 PD-1 (+) cells (right panel) monitored using STAT3 phosphorylation (AlphaLISA) as a surrogate measure of IL-21 activity.

Supplementary Figure 3.

A.

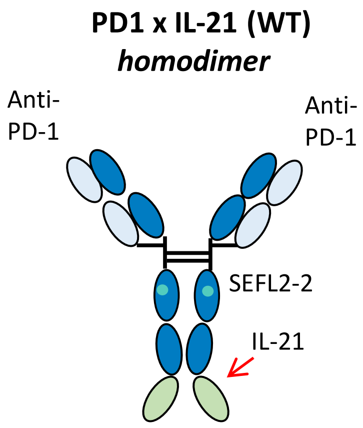

B.

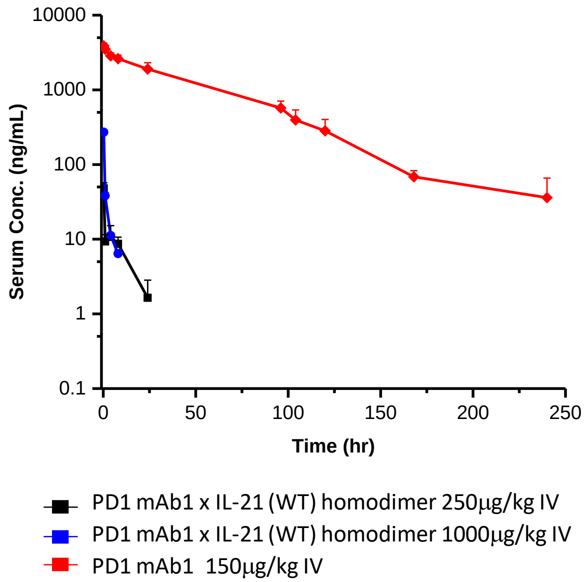

**Intact**	**C_{25min} (ng/mL)**	**AUC (hr*ng/mL)**	**$T_{1/2}$ (hr)**
PD-1 mAb1 x IL21(WT) Homodimer 0.25mg/kg	48 ± 9	189 ± 37	7.5 ± 3.9
PD-1 mAb1 x IL21(WT) Homodimer 1mg/kg	270 ± 22	324 ± 24	2.8 ± 0.2
PD1 mAb1 0.15mg/kg	n/a	n/a	n/a

**Total Fc**	**C_{25min} (ng/mL)**	**AUC (hr*ng/mL)**	**$T_{1/2}$ (hr)**
PD-1 mAb1 x IL21(WT) Homodimer 0.25mg/kg	84 ± 15	408 ± 68	13.5 ± 5.4
PD-1 mAb1 x IL21(WT) Homodimer 1mg/kg	434 ± 46	3530 ± 800	30 ± 12
PD1 mAb1 0.15mg/kg	3890 ± 340	170200 ± 29000	34 ± 17

Supplementary Figure 3. Pharmacokinetic profile of anti-PD-1 x IL-21 WT fusion protein in cynomolgus monkeys. (A) Schematic representation of fusion protein with WT IL-21 fused to C-terminus as homodimer. (B) Mean plasma concentration-time profiles (upper left panel) of WT IL-21 fusion proteins dosed at (single dose) 250 µg/kg or 1000 µg/kg or parent mAb dosed 150 µg/kg (left panel) and parent anti-PD-1 mAb, with summary of pharmacokinetic parameters (right panel). *N = 2* cynomolgus monkeys / group in B.

Supplementary Figure 4.

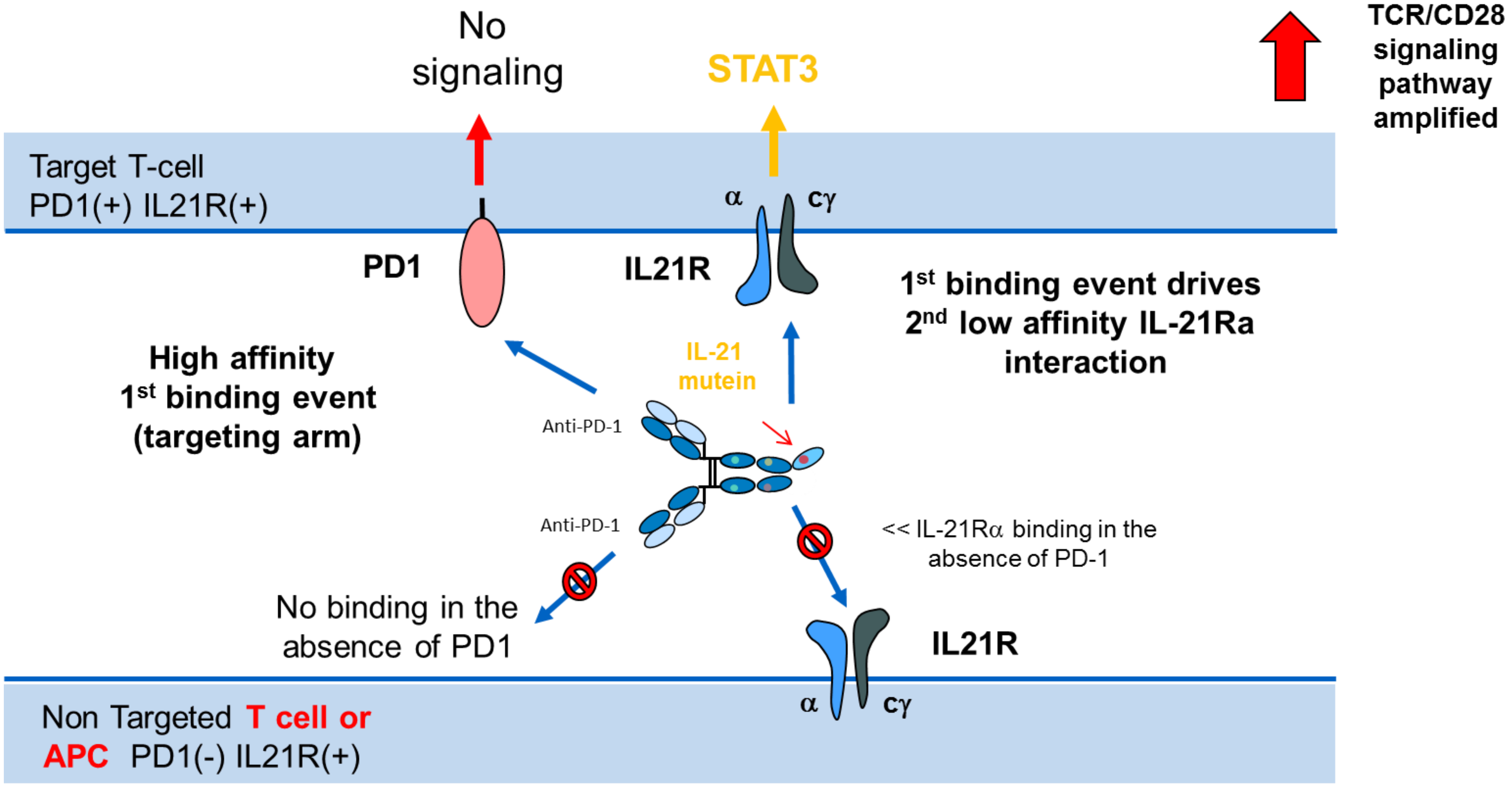

Supplementary Figure 4. Design criteria for anti-PD-1 x IL-21 fusion proteins

Supplementary Figure 5.

A.

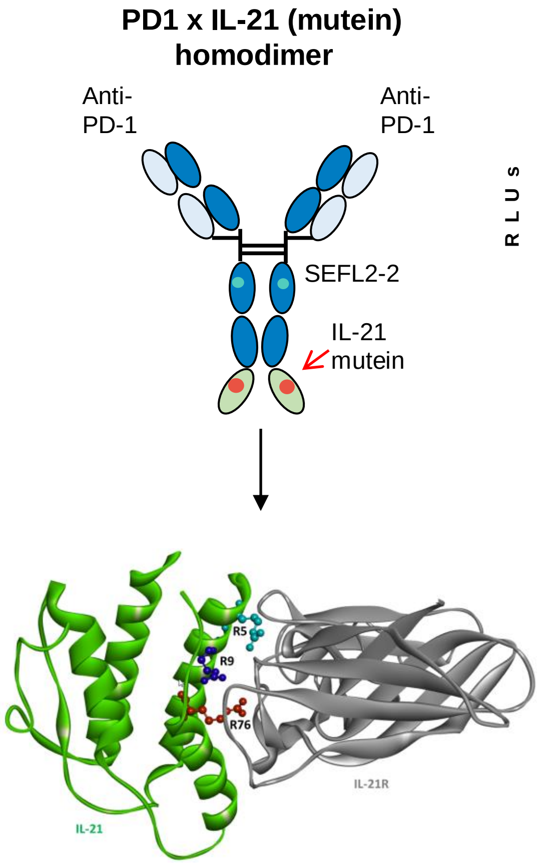

B.

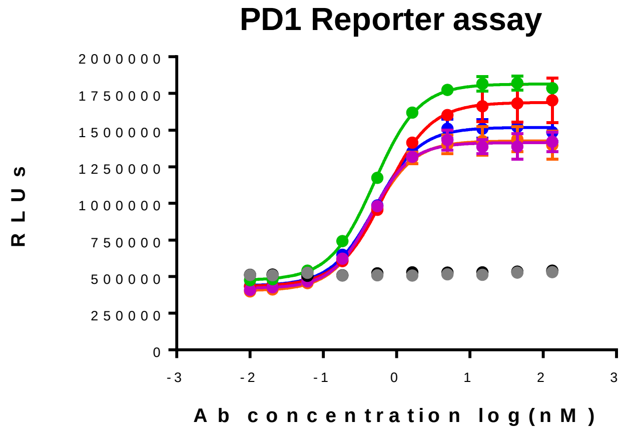

	Interpolated EC50 (nM)
rhIL-21	-
PD1 mAb 2	0.546
PD1 mAb 2 x R5E:R76A homodimer	0.478
PD1 mAb 2 x R9E:R76A homodimer	0.752
PD1 mAb 2 x R5A:R76E homodimer	0.503
PD1 mAb 2 x R5Q:R76E homodimer	0.566

C.

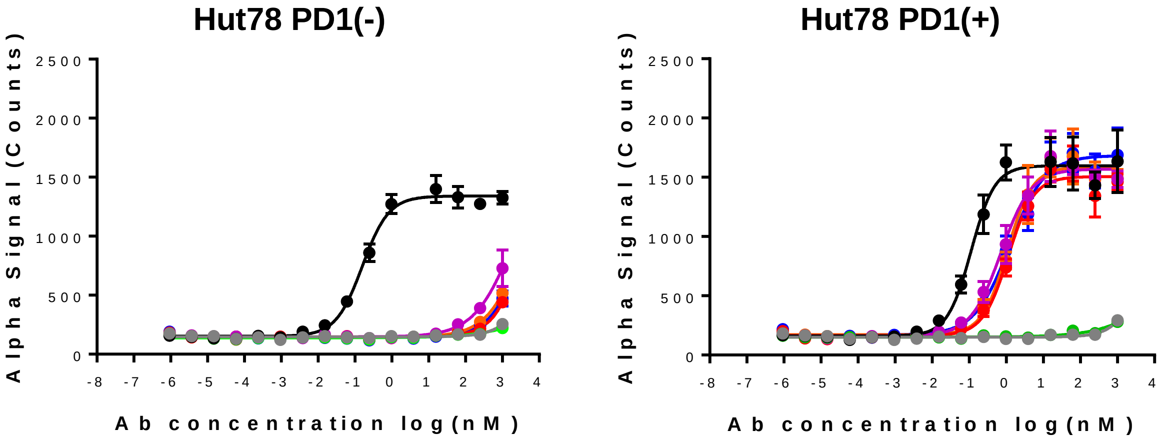

	Hut78 PD1- Interpolated EC50 (nM)	Hut78 PD1+ Interpolated EC50 (nM)
rhIL-21	0.129	0.092
PD1 mAb 2	-	-
PD1 mAb 2 x R5E:R76A homodimer	>1000	0.527
PD1 mAb 2 x R9E:R76A homodimer	>1000	0.778
PD1 mAb 2 x R5A:R76E homodimer	>1000	0.733
PD1 mAb 2 x R5Q:R76E homodimer	>1000	0.897

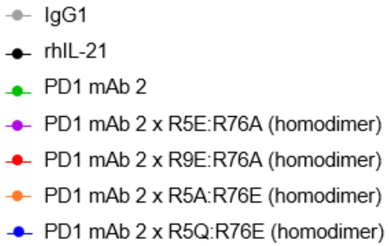

Supplementary Figure 5. Design and characterization of IL-21 variants with dual amino acid substitutions. (A) Schematic representation of fusion protein with dual amino acid IL-21 muteins fused to C-terminus as monomer (upper panel) and key amino acid resides in IL-21 (lower panel). (B) Potency of dual amino acid substitution IL-21 variants fused to anti-PD-1 mAb as monomer and parental mAb in blocking PD-1/L1 interaction monitored using Promega PD-1/L1 bioassay. (C) Activity of dual amino acid substitution IL-21 variants fused to anti-PD-1 mAb as monomer compared to free WT IL-21 in Hut78 PD-1(-) cells (left panel) and engineered Hut78 PD-1 (+) cells (right panel) monitored using STAT3 phosphorylation (AlphaLISA) as a surrogate measure of IL-21 activity.

Supplementary Figure 6.

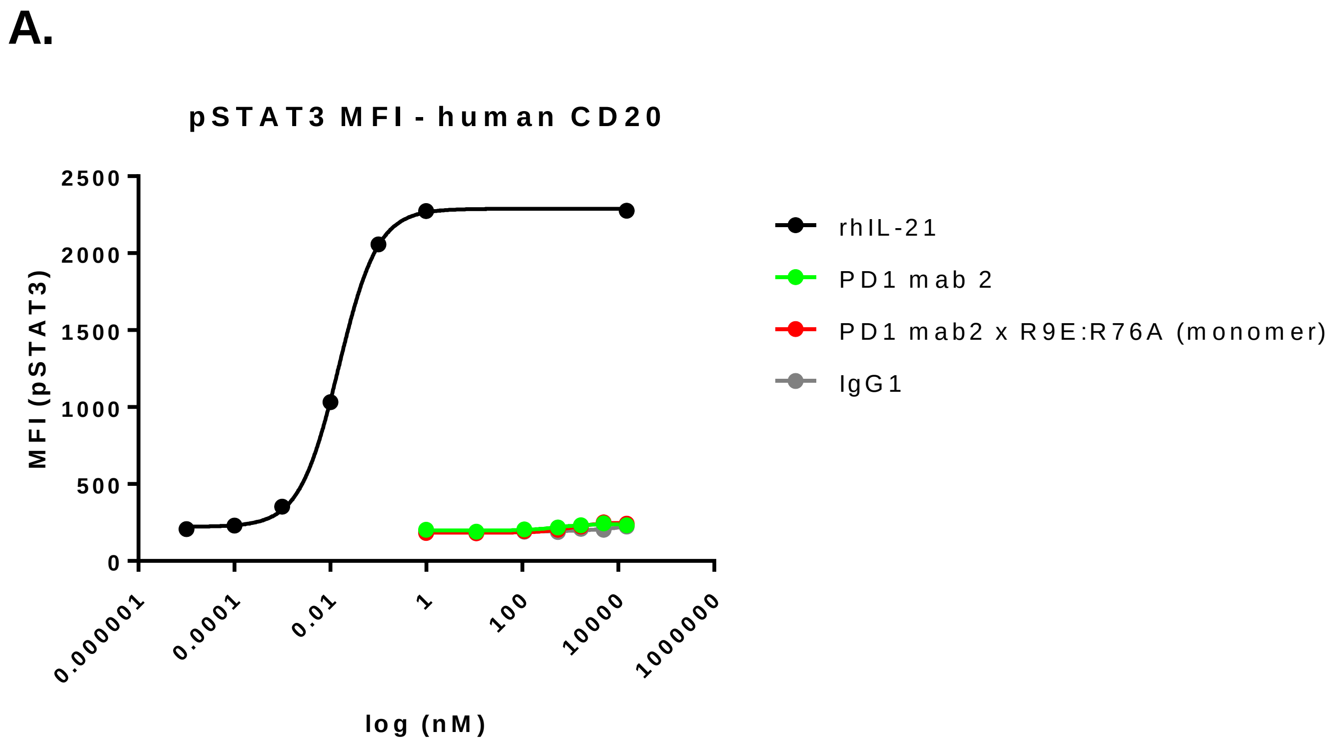

Supplementary Figure 6. Activity of dual amino acid substation variant R9E:R76A on B cells. (A) STAT3 phosphorylation B cells after stimulation for 10 min with free WT IL-21, fusion protein variant R9E:R76A or parental anti-PD-1 mAb, assessed by flow cytometry.

Supplementary Figure 7 for text Table 4.

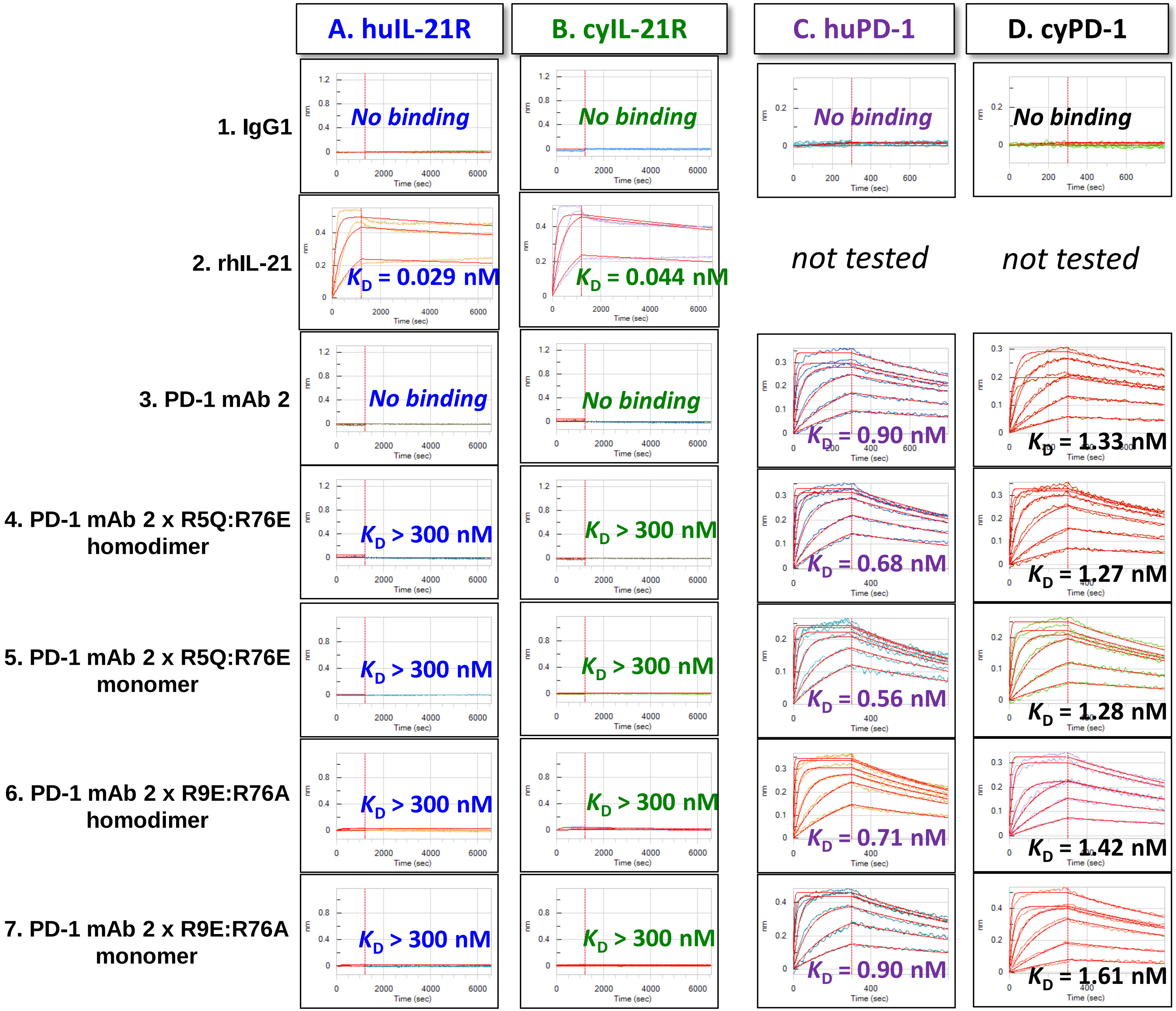

Supplementary Figure 7. ForteBio Octet affinity characterization sensorgrams are shown for seven samples: (1) negative control IgG1, (2) recombinant human IL-21 (rhIL-21) and four test articles (3) parent PD-1 mAb 2, (4) PD-1 mAb 2 x R5Q:R76E Homodimer, (5) PD-1 mAb 2 x R5Q:R76E monomer, (6) PD-1 mAb 2 x R9E:R76A Homodimer and (7) PD-1 mAb 2 x R9E:R76A monomer. The samples were tested for binding to the four soluble receptors (A) human IL-21R (huIL-21R), (B) cynomolgus monkey IL-21R (cyIL-21R), (C) human PD-1 (huPD-1) and (D) cynomolgus monkey PD-1 (cyPD-1). Sensorgrams are shown for the seven samples binding to the four soluble receptors except for rhIL-21 which was only used as a positive control for IL-21R binding and was not tested for PD-1 binding. Each sensorgram shows processed data and corresponding 1:1 model fit as described in methods.

Supplementary Figure 8.

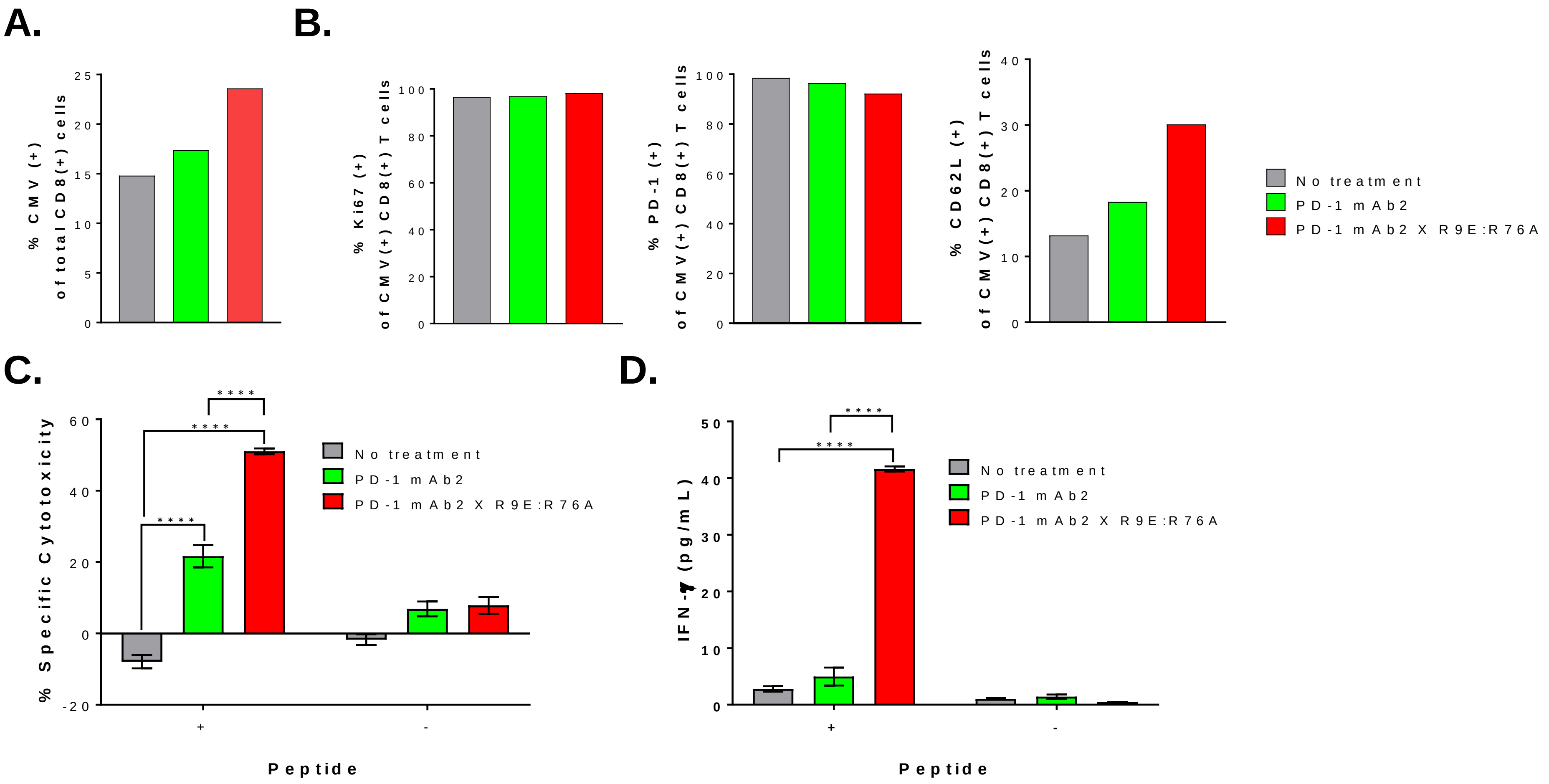

Figure 8. Characterization of PD-1 x IL-21 fusion protein activity on CTLs. CTLs expanded under indicated priming conditions. Five days post priming proportions of (A) CMV antigen specific CTLs and (B) Ki67 (left-panel), PD-1 (middle-panel) and L-selectin (CD62L, right-panel) was analyzed by FACS analysis. CTLs primed under different conditions were isolated and cocultured with peptide pulsed melanoma cells for 36 hrs to examine (C) cytotoxicity against peptide-loaded melanoma cells, determined by measuring luciferase activity and (D) CTL IFN-gamma production. Experiments in (C) and (D) were conducted in triplicates and the error bars represent SEM. P values were calculated using one-way Anova with a Tukey's multiple comparison test. *P* values: ****<*0.0001*, ***<0.001,**<0.01.

Supplementary Figure 9.

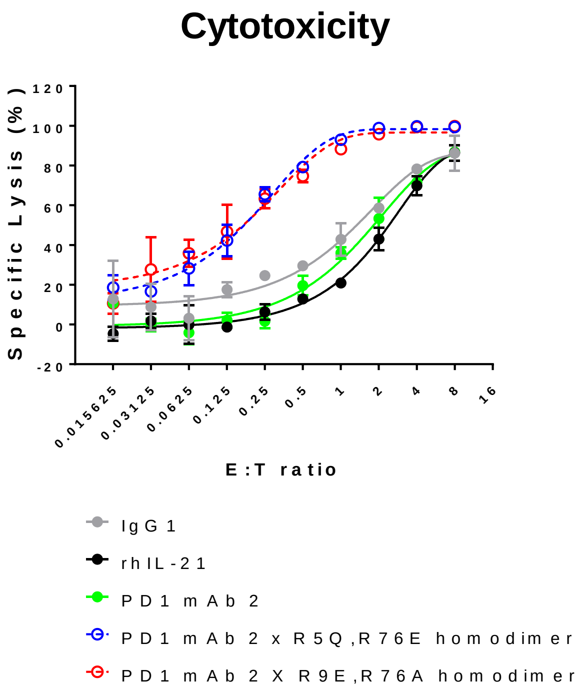

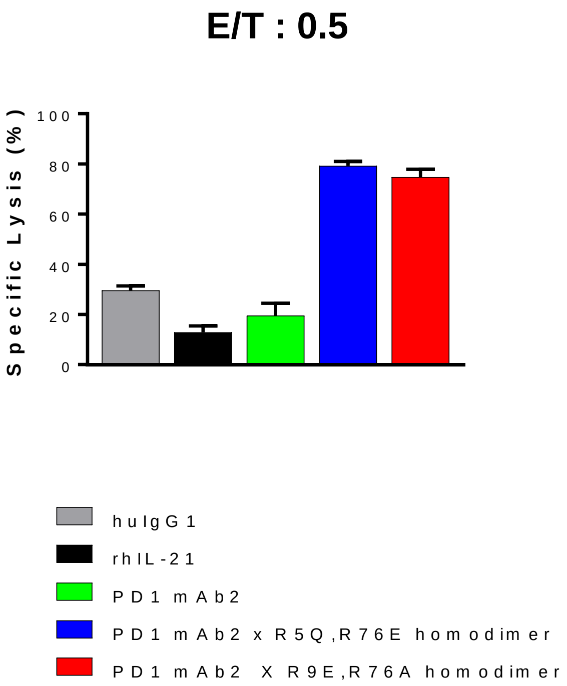

Supplementary Figure 9. Characterization of IL-21 variants with dual amino acid substitutions. Cytotoxicity of CTLs against luciferase labelled melanoma cells was measured after a 7-day treatment of CTLs with CD3/28 and fusion proteins, free- WT IL-21 or parent mAb. Expanded CTLs were co-cultured with peptide pulsed melanoma cells and after 3 days of coculture, cytotoxicity was determined by measuring luciferase activity.

	Hut78 PD-1 (-) Interpolated EC50 (nM)	Hut78 PD-1(+) Interpolated EC50 (nM)	PD-1 reporter Interpolated EC50 (nM)	huIL-21R KD (nM)	muIL-21R KD (nM)	huPD-1 KD (nM)	muPD-1 KD (nM)
IgG1	-	-	-	-	-	-	-
rhIL-21	0.009	0.009	-	0.052	~90	-	-
PD-1 mAb 3	-	-	2.035	-	-	3.4	-
PD-1 mAb 3 x R9E:R76A monomer	>1000	1.97	1.613	>300	-	2.6	-

Supplementary Table 1. Summary of *in vitro* attributes of anti-PD-1 x IL-21 double *muteins*
